# A Case of Cholecystotomy, with Recovery

**Published:** 1888-04

**Authors:** 


### AN INTERESTING CASE OF CHOLECYSTOTOMY, WITH RECOVERY.

On February 14, M. Polaillon reported to the Academy of Medicine, of Paris, a case of cholecystotomy performed by M. Terrillon, and followed by recovery.

The patient was a woman, æt. 24, who had had no previous liver trouble until she noticed a tumor in the right side, which was easily felt below the free border of the ribs by Terrillon. It was very large and extended to a point below the umbilicus. The skin over the tumor was normal, the umbilical cicatrix regular, and there were no enlarged subcutaneous veins. Palpation showed the tumor to be as large as the head of a fœtus, tense, elongated vertically, smooth, and very movable transversely. It did not appear to follow the respiratory movements. Its extent was easily made out by percussion. Its dullness was co-extensive with that of the liver; in other respects percussion of the abdomen was normal. There was no icterus, no pain, and no serious inconvenience of any kind, though there was some dyspepsia and emaciation. The urine was normal.

It was evident that the tumor was connected with or adherent to the liver. No exploratory puncture was made, but Terrillon determined to make an exploratory laparatomy. The operation was performed on November 23, 1886. A median vertical incision was made, about four cm. long, and afterwards enlarged to eight cm. When the abdomen was opened the lower border of the liver was easily seen, as it was lower than normal. Co-extensive with the lower border and attached to the inferior surface was an elongated tumor, which descended very low, had bluish walls, was very fluctuant, and of a cystic appearance. The exploring finger showed that the tumor was the distended gall-bladder. Puncture with a capillary trocar gave issue to a liquid as clear as water, but a little thicker, the last drops of which were of a milky whiteness. It was then found that the bladder contained a calculus as large as a cherry, The bladder was then drawn partly out of the abdomen, sutured to the abdominal wound, and opened. Its walls were very vascular, and hæmorrhage was guarded against by means of forceps, and the cavity was explored.

The calculus first found was easily extracted, though it was adherent to the bladder by a thin filament. The exploring finger then found at the summit of the bladder a second calculus imbedded in the mucous membrane. It was so deeply imbedded that at the distance it was from the opening it was impossible to detach it; the bladder was drawn farther out, so as to lessen the depth of the cavity, when the stone was removed in fragments with the forceps.

Having resected a portion of the base of the bladder, the operator sutured it to the abdominal wall, making a biliary fistula in which two drainage-tubes were placed; a dressing was then applied. In about a month the fistula was so far closed that it admitted only a very small filiform bougie. Bile flowed abundantly from this orifice, generally between 9 p. m. and 2 a. m.—about four hours after the last meal of the day. Two cauterizations with the thermo-cautery caused the fistula to close completely two months after the operation. Meanwhile the patient had gained flesh, and had completely recovered.

In commenting upon the case M. Polaillon says that generally the most favorable incision for cholecystotomy is one along the external border of the rectus muscle of the right side. But in order to have more room one must sometimes add to this a more or lese transverse incision a little below the border of the costal cartilages. Terrillon's incision, however, was primarily intended for an exploratory laparotomy. Opening the gall-bladder may cause hæmorrhage, which must be controlled by means of hæmostatic forceps, or to extravasation of bile, which must be prevented at all hazards by drawing the bladder out and protecting the peritoneum by aseptic sponges. Suture of the abdominal wall completes the operation in cases in which there is no calculus. A biliary fistula is established, and the most pressing danger, intra-peritoneal rupture of the gall-bladder, is averted.

When there are calculi, these must be removed, and the operator must remove all obstructions from the cystic and common duct, so that the bile may escape normally into the intestine. Extraction of the calculi is sometimes a serious difficulty, and

Fauconneau-Duferesne recommends diminishing their size by crushing with a small lithotrite. Sometimes it is very difficult to tell during the operation whether all the calculi have been removed, and if all are not removed the operation does not accomplish its end.

The mortality of cholecystotomy, excluding the fatal cases not due to the operation, is about 16 per centum. It is then a serious operation, but it must be borne in mind that it is performed on patients whose lives are actually in danger. Polaillon advises that the operation be always preceded by an exploratory puncture, so as to confirm the diagnosis. The Academy adopted the conclusions drawn by Polaillon.—*Bulletin de l'Académie de Médicine*, No. 7, 1888.

